# Highly Aggressive Intraparenchymal Solitary Fibrous Tumor of the Lung with Distant Metastasis: A Case Report and Review of CT and PET/CT Findings

**DOI:** 10.3390/reports8020078

**Published:** 2025-05-23

**Authors:** Jeong Joo Woo, Jin Kyung An

**Affiliations:** Department of Radiology, Nowon Eulji University Hospital, Eulji University School of Medicine, Seoul 01830, Republic of Korea

**Keywords:** solitary fibrous tumor, lungs, computed tomography, PET/CT

## Abstract

**Background and Clinical Significance:** Solitary fibrous tumors (SFTs) arising from the lung parenchyma without any relation to the pleura are rare. **Case Presentation:** We report a case of highly aggressive intraparenchymal SFT of the lung in a 52-year-old woman with rapid distant metastasis to the brain, lungs, and bones within one year post-operation. Chest computed tomography (CT) showed a 5.5 cm-sized, round, but partially lobulated mass with ambiguous enhancement in the right upper lobe. Positron emission tomography/computed tomography (PET/CT) demonstrated strong homogeneous FDG uptake. Unfortunately, the patient succumbed to the disease within one year of diagnosis. **Conclusions:** Among intrapulmonary SFT, the cellular variant may appear as a cystic mass due to accompanying hemorrhage, coagulation necrosis, and myxoid degeneration. In the absence of mediastinal metastatic adenopathy, it can be mistaken for a benign cystic mass, making PET/CT findings a crucial tool for suggesting a malignancy. Furthermore, as cellular-type intrapulmonary SFT can exhibit aggressive distant metastasis, understanding the CT and PET/CT findings in this condition is essential for accurate diagnosis and treatment planning.

## 1. Introduction and Clinical Significance

Solitary fibrous tumors (SFTs) were first described as rare mesenchymal neoplasms by Wagner in 1870. In 1931, Klemperer and Coleman characterized them as pleural lesions of mesothelial origin [1,2,3]. Solitary fibrous tumors are now considered soft tissue neoplasms of pluripotent fibroblastic or myofibroblastic origin and are ubiquitous in nature [2].

A solitary fibrous tumor is a neoplasm that evolves and exhibits variable histologic characteristics, resulting in variable imaging findings. While most cases are benign, approximately 10–20% may exhibit malignant behavior, making the prediction of malignancy based on imaging features critical for treatment planning. It is essential to comprehensively assess CT and PET/CT findings.

## 2. Clinical Presentation

**Figure 1:**
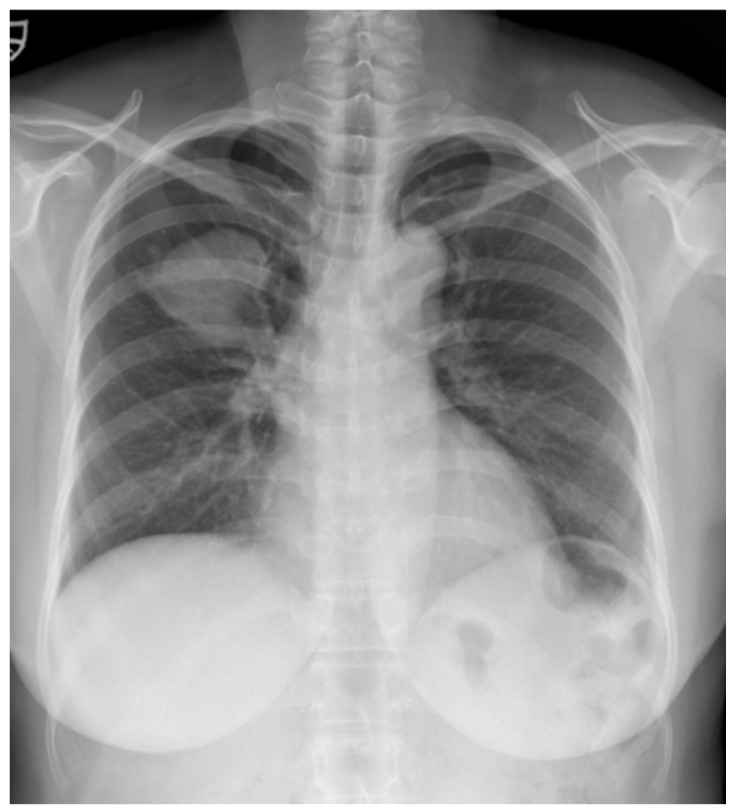
A chest radiograph at the initial visit showed a well-defined mass-like opacity with a partially lobulated lateral edge in the right upper zone.

**Figure 2:**
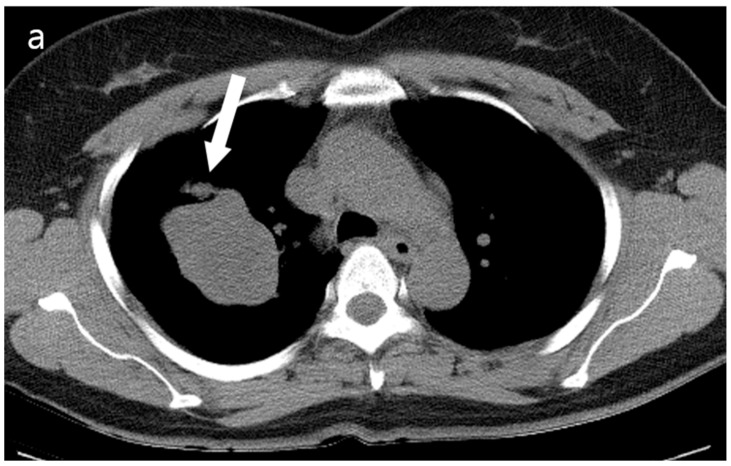

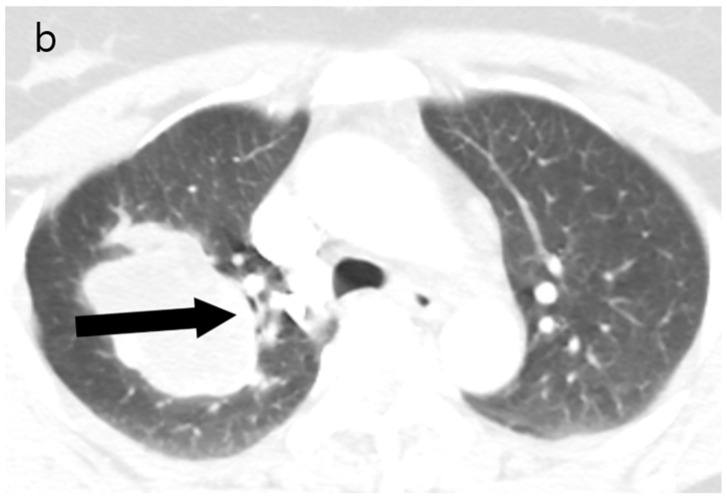
(**a**) Unenhanced chest CT showed a homogeneous hypodense mass (5.6 cm in diameter) in the right upper lobe (white arrow). The mass had a well-defined shape with a primarily smooth, but partially beak-like lobulated margin. (**b**) There was splaying of the anterior and posterior segmental bronchi (black arrow) from the mass effect.

**Figure 3:**
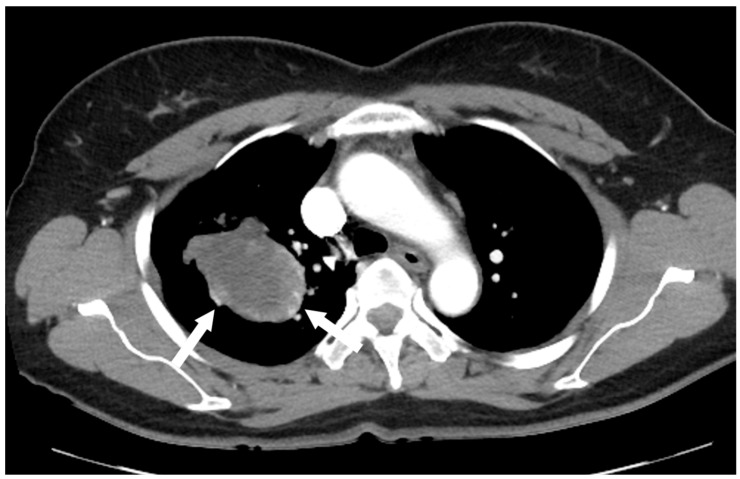
Contrast-enhanced chest CT revealed a poorly enhancing mass resembling a cystic lesion, but with strong nodular or curvilinear enhancement along the periphery of the mass.

A 52-year-old woman was admitted to our hospital with a three-month history of cough and sputum, along with a one-month history of hemoptysis. Her medical history was unremarkable, and she was a nonsmoker. The laboratory results were within normal limits. Chest radiography revealed a well-defined mass-like opacity with a partially lobulated lateral edge in the right upper zone (Figure 1). Unenhanced CT showed a homogeneous hypodense mass (Hounsfield Unit: 20) measuring approximately 56 × 44 × 42 mm  in diameter, with well-defined, mostly smooth, but partially lobulated margins in the right upper lobe (Figure 2a). The mass effect caused splaying of the anterior and posterior segmental bronchi, unlike bronchial obstruction typically observed in lung cancer (Figure 2b). Contrast-enhanced CT (CECT) revealed a predominantly poorly enhancing mass resembling a cystic lesion with a small but strong nodular or curvilinear peripheral enhancement (Figure 3). No mediastinal adenopathy was observed.

### 2.1. Differential Diagnosis

The differential diagnosis included an unusual tumor with massive necrosis or cystic degeneration, such as sarcoma or SFT, and congenital cystic lesions, such as bronchocele. Lung cancer with necrotic changes was considered less likely due to the bizarre shape, absence of mediastinal adenopathy, and poor enhancement within most of the masses.

### 2.2. Investigations

**Figure 4:**
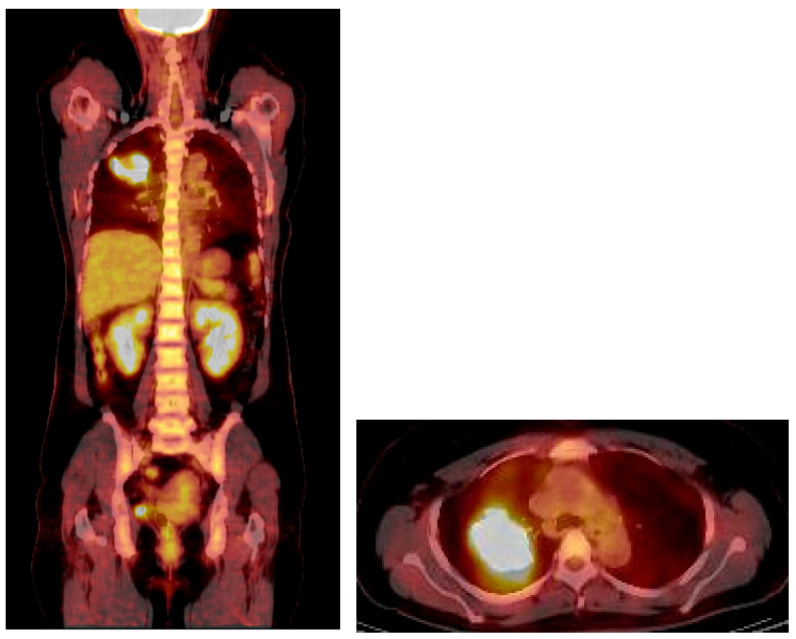
PET/CT revealed intense FDG uptake in the corresponding mass in the right upper lung, with no evidence of extrathoracic involvement.

An F-18 FDG PET/CT torso scan was performed four days after the initial CT examination to assess the metabolic activity of the mass. Surprisingly, PET/CT revealed intense FDG accumulation in the mass, with a maximal standardized uptake value (SUVmax) of 13.4, and no other abnormal lesions were detected throughout the body (Figure 4). Since the brain was not included in PET/CT, brain MRI was performed three days after the PET scan. There were no particular abnormalities on the brain MRI.

### 2.3. Outcome and Follow-Up

**Figure 5:**
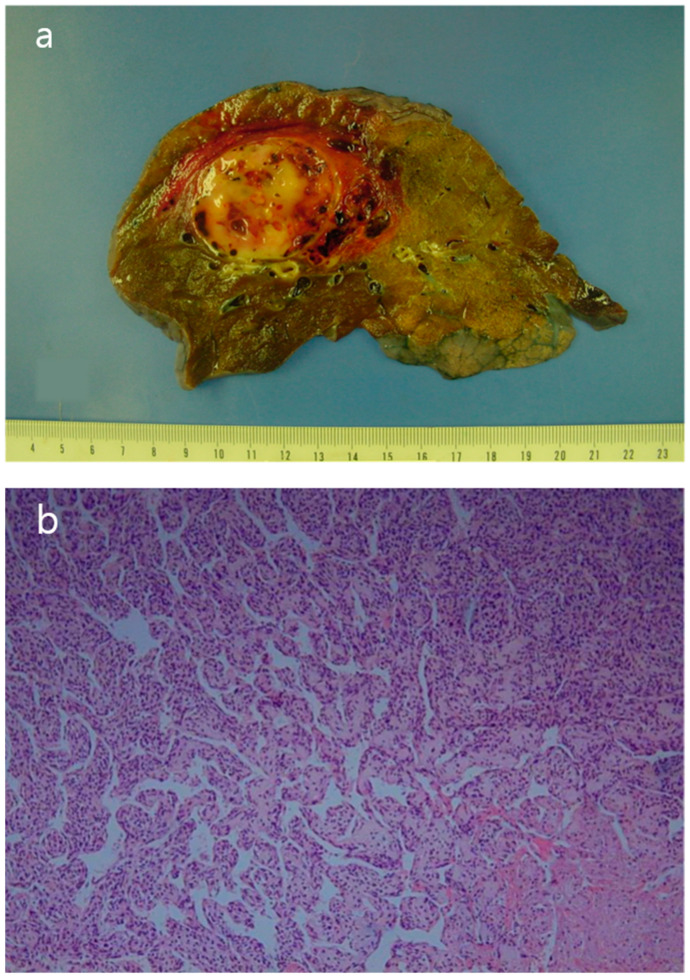

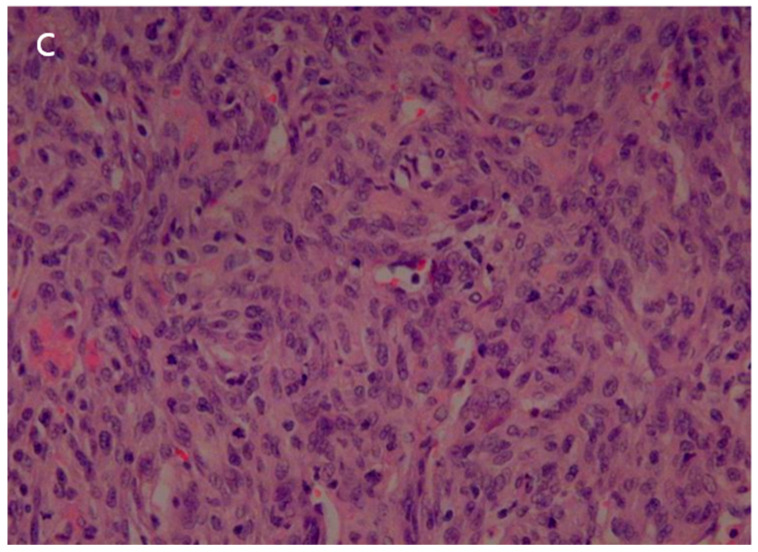
(**a**) The surgical specimen revealed a well-circumscribed, pale-yellow, soft mass (5.5 cm in diameter) with hemorrhage. (**b**) Microscopic imaging of the tumor showed a hemangiopericytic pattern and areas of coagulation necrosis (hematoxylin and eosin [HE] × 100). (**c**) Numerous mitotic figures and marked nuclear pleomorphism were observed in high-grade areas (HE × 400).

The patient underwent a right upper lobe lobectomy. Gross examination showed a well-circumscribed, pale yellow, soft tumor with hemorrhage, measuring 5.5 × 5.0 cm (Figure 5a). Histopathological analysis revealed a hemangiopericytic growth pattern with areas of coagulation necrosis (Figure 5b). Numerous mitotic figures and marked nuclear pleomorphisms resembled high-grade pleomorphic sarcomas (Figure 5c). Immunohistochemically, the tumor cells were focally positive for CD34 and negative for epithelial markers, such as cytokeratin and EMA (epithelial membrane antigen). Neoplastic cells were negative for desmin, myoglobin, Bcl-2, calretinin, and HMB-45. The immunohistochemical results favored the diagnosis of a malignant solitary fibrous tumor.

**Figure 6:**
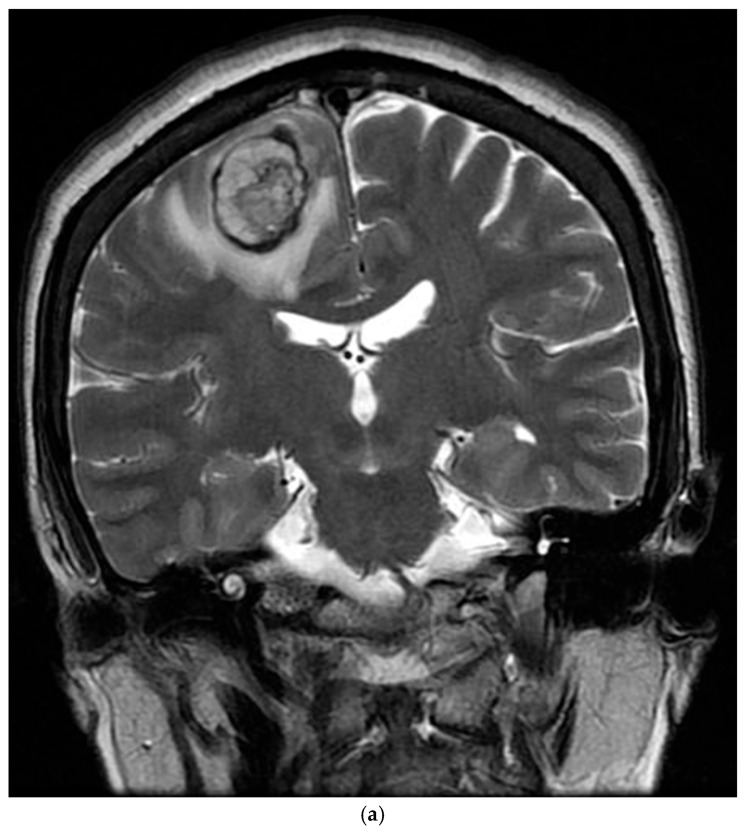

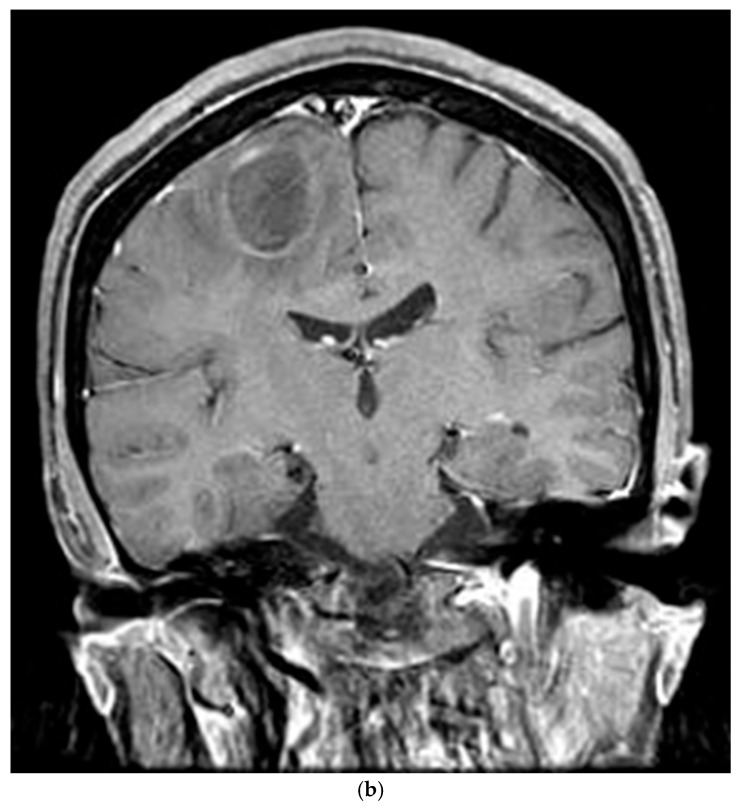
Three-months postoperative coronal (**a**) T2 and (**b**) enhanced T1 images revealed a hemorrhagic rim-enhancing mass with perilesional edema in the right frontal lobe.

The patient’s postoperative period was uneventful, and she was discharged and subsequently administered chemotherapy. However, three months later, the patient was readmitted with left-sided hemiparesis. Brain MRI revealed a 25 mm T2-high signal and T1-low signal intensity mass with perilesional edema and faint peripheral enhancement in the posterior portion of the right superior frontal gyrus (Figure 6). Stereotaxic biopsy of the brain mass confirmed metastasis from the primary lung tumor. The metastatic brain tumor with hemorrhage was surgically removed, and the patient was started on chemotherapy and radiotherapy.

Nine months after the initial surgery, extensive metastatic lesions were detected in the lungs, adrenal glands, ribs, and erector spinae muscle. One month later, she was admitted to the emergency room with an altered mental status. Enhanced brain CT and MRI showed metastatic hemorrhagic tumors in the left temporal and right parietal lobes. Her condition continued to deteriorate, and two weeks later, she succumbed to respiratory arrest. This study was approved by the Institutional Review Board of our hospital (IRB No. Eulji 2023-12-015).

## 3. Discussion

Whether SFT are derived from mesothelial or mesenchymal cells is controversial. Most investigators accept that SFT are mesenchymal tumors arise from dendritic stromal cells expressing the CD34 antigen, and can occur in any body part. Recent updates in tumor classification have consolidated pleural-localized fibrous tumors, intrapulmonary masses exhibiting SFT features, and intrapulmonary masses showing hemangiopericytoma (HPC) features into a single entity, such as intrathoracic SFT [2,3,4,5]. Pulmonary HPCs reported before the nomenclature change are classified as cellular SFTs, a rare and more aggressive variant with different radiological features compared to the more common and benign pleural SFTs. Differentiating between these tumors is essential for treatment planning.

Malignant SFTs account for only 10–20% of cases. Indicators of malignancy include large tumor size (>5 cm), nuclear pleomorphism, increased cellularity, and an elevated mitotic index (>4 mitoses per 10 HPF) [2,3,6]. Imaging features suggestive of malignancy include larger size (>15 cm), compression of adjacent structures, central necrosis, ipsilateral pleural effusion, and infiltrative chest wall invasion [7,8,9,10]. In this case, the initial CT scan revealed a large mass in the lung that appeared to be a cystic lesion and did not show significant lymph node involvement, suggesting a more benign condition. However, the PET/CT demonstrated intense FDG accumulation, indicating the possibility of malignancy. As seen in this case, when coagulation necrosis and myxoid degeneration are present, the tumor may present as a non-enhancing cystic mass on a CT scan, which can lead to misdiagnosis. In such cases, evaluation of metabolic activity using PET/CT is crucial for accurate characterization.

The patient exhibited a highly aggressive course, with brain metastasis occurring just three months post-surgery. Such rapid progression and brain metastases have rarely been reported. Extensive metastases to the contralateral lung, ribs, and brain developed within a year, highlighting the aggressive nature of the tumor.

Most intrathoracic SFTs show low-to-moderate FDG uptake in the mass on PET/CT. However, the tumor in this case exhibited high cellularity, which was suspected to have contributed to the high uptake of FDG. Although some studies have suggested that PET is not useful for differentiating between benign and malignant SFTs, avid uptake is interpreted as malignant potential.

Complete surgical resection is the treatment of choice and a wide excision margin  is recommended to prevent local spread. Patients with metastatic and malignant SFT may require pre-surgical or post-surgical radiation and chemotherapy, although they often show resistance to these modalities. Despite surgical resection and postsurgical chemotherapy, the patient underwent a highly aggressive course. Adjuvant chemotherapy should be considered when malignancy is suspected.

## 4. Conclusions

The cellular variant of solitary fibrous tumors (SFT) in the lung, without any relation to the pleura, can pose a diagnostic challenge for radiologists because CT findings may not be differentiated from other lung masses or cystic lesions, especially when coagulation necrosis or myxoid degeneration is present. The possibility of malignant SFT should be considered when a predominantly non-enhancing, irregular-shaped lung mass shows subtle peripheral enhancement, even without lymphadenopathy or pleural metastasis. PET/CT plays a crucial role in assessing the malignant potential of SFT and in treatment planning.

## Data Availability

The original contributions presented in this study are included in the article. Further inquiries can be directed to the corresponding author.
